# How well does TOKS identify patients with severe sepsis or septic shock?

**DOI:** 10.1186/1757-7241-21-S2-A28

**Published:** 2013-09-09

**Authors:** Anette Tanderup, Merete Storgaard, Annmarie Lassen

**Affiliations:** 1 Department of Geriatric, Odense University Hospital, Denmark; 2 Department of Infectious Medicine, Aarhus University, Denmark; 3 Department of Emergency Medicine, Odense University Hospital, Denmark

## Background

Several scoring system have been developed with the aim to identify clinical deterioration among hospitalized patients and allocate resources in accordance with the degree of deterioration - most without validation. The aim of the present study was to describe to which degree the system "Tidlig Opsporing af Kritisk Sygdom" (TOKS) is able to identify patients who either have or develop severe sepsis or septic shock within 24 after arrival to hospital.

## Methods

A retrospective descriptive study of patients hospitalized with community acquired severe sepsis or septic shock. Patients were identified based at discharge diagnosis ((IDC10 code A40.0-A41.9). Based at a manual evaluation of all patient records patients were included if they within the first 24 hours after arrival to the hospital fulfilled predefined criteria for severe sepsis or septic shock. Vital values registered at arrival to the hospital were identified and used for the present analysis. TOKS score is based at scores for respiratory frequency, saturation, systolic blood pressure, pulse rate, consciousness and temperature. The score range from 0 to 21 with an indication of need for a doctoral evaluation if the score is 3 or higher.

## Results

335 patients were discharged with a diagnosis of sepsis. 212 fulfilled the criteria for severe sepsis or septic shock within the first 24 hours of hospitalization. One hundred and six (50%) were male, mean age 70.6 years (SD 14.7, range 24.0-96.6 years), 103 (49%) had septic shock. Median TOKS score at arrival was 4 (range 0-13). 10/212 (5%) had TOKS=0, indicating no need for measurements of vital values the next 24 hours, 20/212 (9%) had TOKS=1, indicating measurements of vital values every 8 hours, 13/212 (6%) had TOKS=2 indicating control of vital values after one hour, 66/212(31%) had TOKS 3-4 indicating need for evaluation by a junior doctor and 103/121 (49%) had a TOKS score≥5 indicating need for urgent specialist evaluation.

## Conclusion

14% of the patients who develop severe sepsis or septic shock within 24 hours after arrival to the hospital had a TOKS score at arrival indicating a need for control of vital values every 8 hours or less.

